# 

**Published:** 1890-05

**Authors:** 


### CONTENTS.

COMMUNICATIONS.

Evolution in Dentistry ........ 213
Affections Liable to Occur to the Alveolar Process ........ 218
Rhino-Plasty ........ 221

PROCEEDINGS

The Forty-sixth Annual Meeting of the Mississipi Valley Association of Dental Surgeons ........ 225

SELECTIONS.

Hyderabad Chloroform Commission ........ 234
Notes and Comments—A Curious Hypnotic Test ........ 239
Doctors and Dentists with American Diplomas ........ 240
Do Heads Grow with Advanced Age? ........ 241
American Medical Editors' Association ........ 242
Guy's Hospital Dental School ........ 242
Dental College Commencements ........ 243

CORRESPONDENCE.

Trouble in the Camp ........ 253

EDITORIAL.

Seventh District Dental Society—Ohio ........ 254
International Medical Congress ........ 255
Dental Society Work ........ 257
Should Physicians be Illiterate? ........ 258
Iowa State Dental Association ........ 259
American Medical Association ........ 260
A New Medical Dictionary ........ 260
Dental Anatomy ........ 261
The Dental Protective Association ........ 262
Northern Ohio Dental Association ........ 264

